# Dataset on adsorption of methylene blue from aqueous solution onto activated carbon obtained from low cost wastes by chemical-thermal activation – modelling using response surface methodology

**DOI:** 10.1016/j.dib.2019.104036

**Published:** 2019-05-23

**Authors:** Danial Nayeri, Seyyed Alireza Mousavi, Mahsa Fatahi, Ali Almasi, Faranak Khodadoost

**Affiliations:** a Department of Environmental Health, Faculty of Health, Research Center for Environmental Determinants of Health (RCEDH), Kermanshah University of Medical Sciences, Kermanshah, Iran; b Social Development and Health Promotion Research Center, Kermanshah University of Medical Sciences, Kermanshah, Iran; c Student Research Committee, Kermanshah University of Medical Sciences, Kermanshah, Iran

**Keywords:** Methylene blue, Adsorption, Response surface methodology, Waste corn

## Abstract

The aim of this study was to produce activated carbon derived from corn stalk (AC-CS) with suitable characteristics as inexpensive, nontoxic adsorbent with good efficiency for elimination of Methylene Blue (MB) as cationic dye from aqueous solution in batch adsorption process. The morphology and functional groups of adsorbent were characterized by SEM and FTIR in this dataset. In addition, the influence of MB concentration, pH, adsorbent dosage, and contact time on the removal of dye using AC-CS was tested by central composite design (CCD) under response surface methodology (RSM). Based on results, the parameters adsorbent dose and initial dye concentration for this investigation play an important role in the adsorption studies of methylene blue. The experimental values were in good agreement with the model predicted values also the results of the study showed that maximum absorbance efficiency at initial concentration of 10 mg/l, absorbent dose of 1.4 g, contact time of 50 min and pH 11 was 90%.

**Table:** Specifications table

Subject area	Environmental sciences
More specific subject area	Adsorption
Type of data	Tables and figures
How data was acquired?	In this study, AC-CS was prepared and used for MB removal from aqueous solution. Prepared activated carbon characterized by FT-IR, and SEM techniques. Response surface methodology (RSM) was used to modelling and optimizing based on four independent variables, including adsorbent dosage, initial concentration of MB, pH and contact time.
Data format	Raw, analyzed
Experimental factors	The main independent factors including initial pH of solution, contact time, initial day concentration, adsorbent dosage and the main dependent factor was final concentration of MB.
Experimental features	The main aims of this study were to prepare an activated carbon from corn stalk, study the efficiency removal of MB from aqueous solution using AC-CS, modeling and optimization of process
Data source location	Kermanshah University of Medical Sciences, Kermanshah, Iran
Data accessibility	Data are summarized in this article
Related research article	S.A. Mousavi, M. Mehralian, M. Khashij, S. Parvaneh, Methylene Blue removal from aqueous solutions by activated carbon prepared from N. microphyllum (AC-NM): RSM analysis, isotherms and kinetic studies, Glob. NEST J, 19 (2017) 697-705

**Table:** 

**Value of the data** • The achieved data are useful for preparing AC from corn stalk that has ability to remove MB from water and wastewater • The obtained data introduce a cost effectiveness adsorbent that can be useful for future similar studies • The modeling and optimization of data can help researchers to predict the effect of studied variables at different values

## 1 Data

**Fig. 1:**
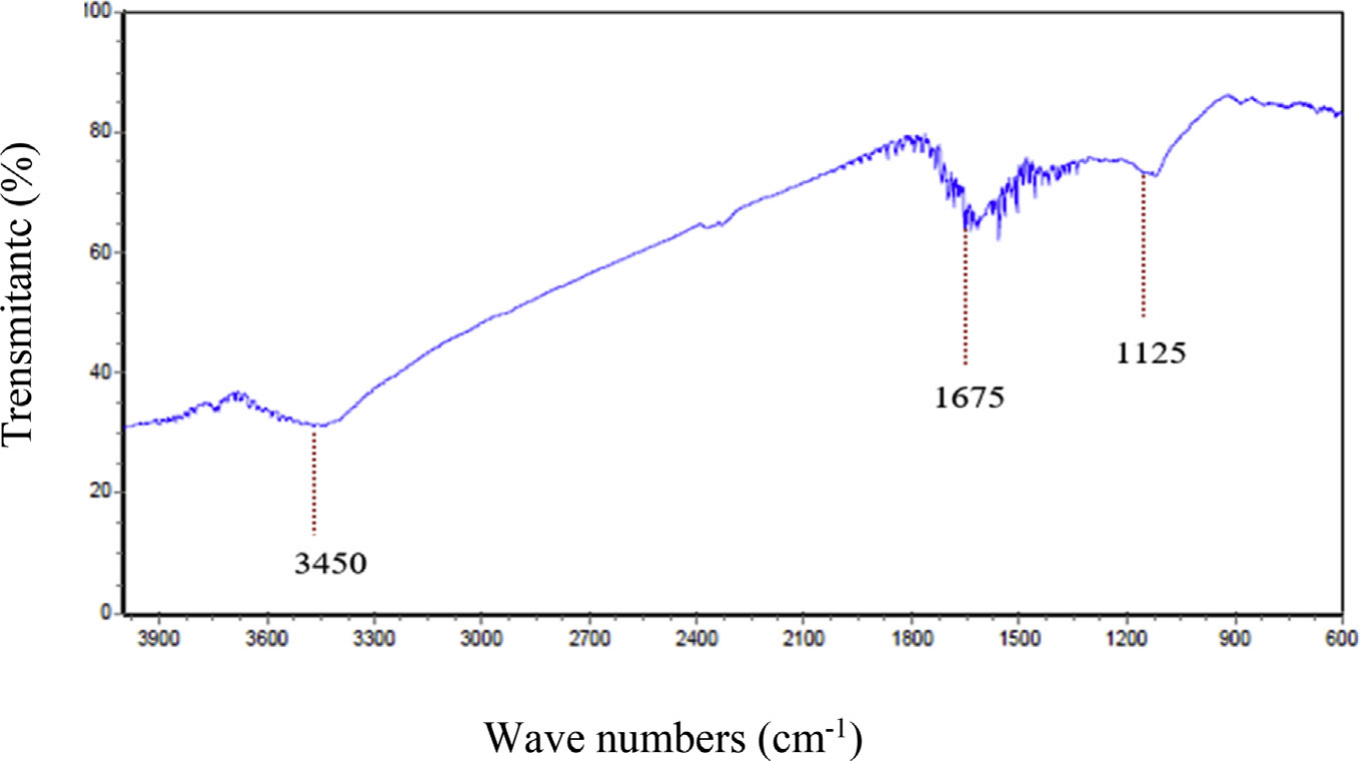
FTIR spectrum.

**Fig. 2:**
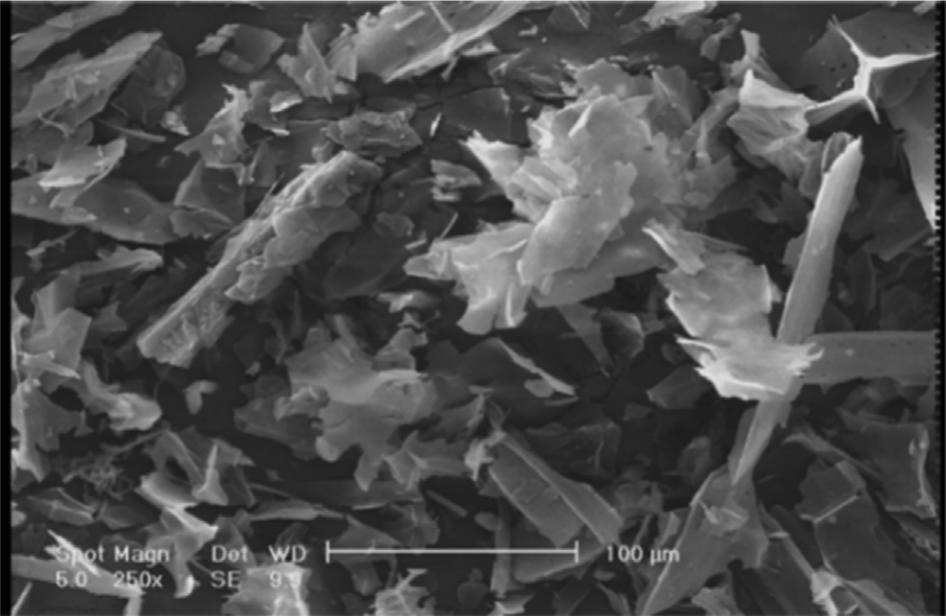
SEM images of prepared activated carbon from corn waste.

**Fig. 3:**
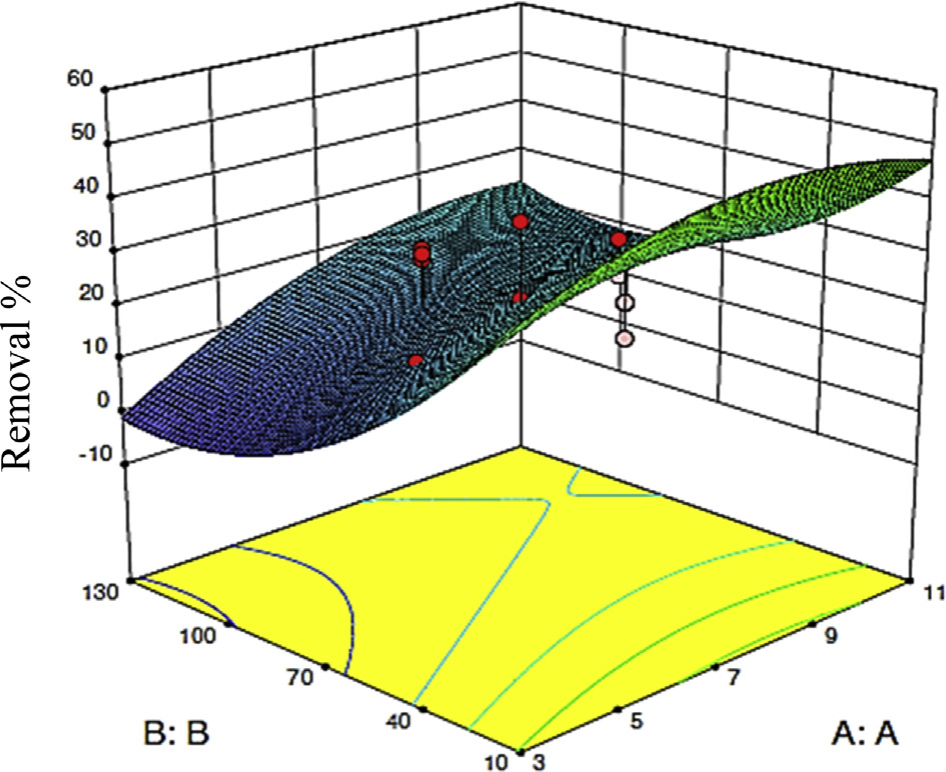
Response surface plots showing the effect of pH and initial concentration on MB removal (Dosage = 0.8 g/l and Contact time = 30 min).

**Fig. 4:**
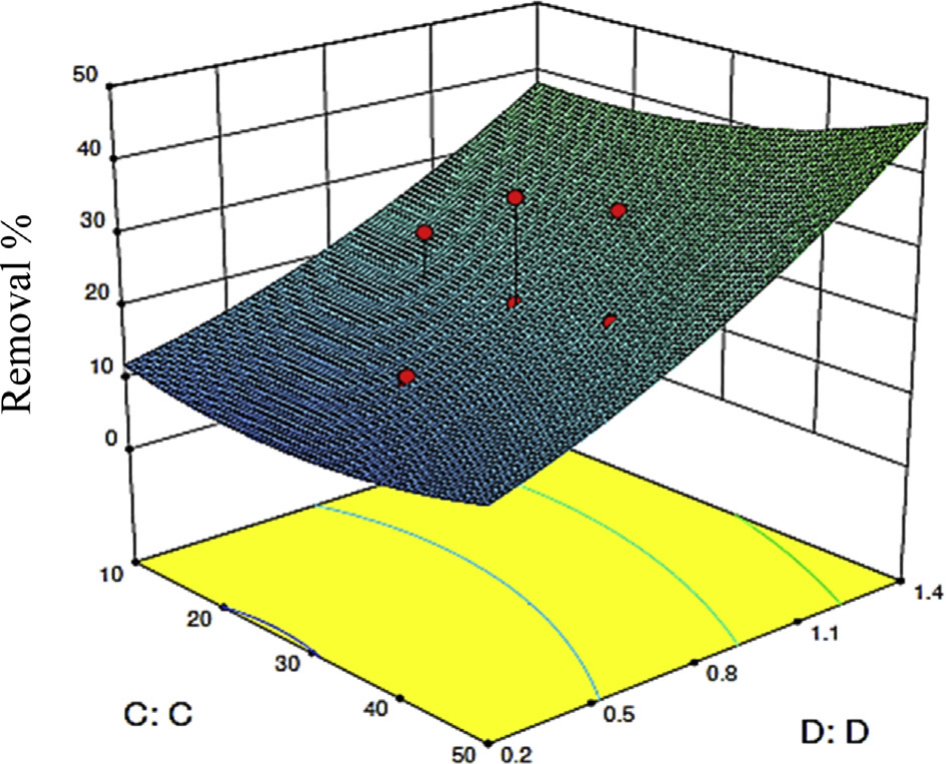
Response surface plots showing the effect of dosage and contact time on MB removal (pH 7 and initial concentration = 70 mg/l).

This dataset contains experimental design and results of CCD using DOE software version 8 according to Table 1. The results of chemical and physical adsorbent characterization represented in Fig. 1 and Fig. 2. The FT-IR spectra of the AC-CS according to Fig. 1 confirm different functional groups; the –OH stretching vibration mode of hydroxyl functional groups located at about 3450 cm^−1^ [1], [2], the peak at about 1675cm^−1^ related to the C O stretching vibration of lactonic and carbonyl groups [2], [3], and the band located at about 1125 cm^−1^ attributed to carboxylic groups [3], [4]. The surface physical morphology of the AC-CS according to scanning electron microscopy (SEM) technique (Fig. 2) shows an irregular and porous structure. The results of the statistical analysis confirmed the adequacy of the model (p < 0.0001), and the significance of independent factors; A: pH, B: MB concentration (mg/l), C: Contact time (min), and D: Adsorbent dosage (g/l), which are <0.0001, <0.0001, <0.0001, 0.0162, <0.0001, respectively. Analysis of variance (ANOVA) and optimized values of parameters on the MB adsorption by AC-CS reported in Table 2 and Table 3, respectively. The response surface analysis in Fig. 3 and Fig. 4 show the effect of the main parameters, namely, pH, MB concentration (mg/l), contact time (min), and adsorbent dosage (g/l), on the efficiency of dye removal during adsorption.

**Table 1:** Experimental conditions and results of CCD.

Run No.	A: pH	B: initial MB concentration (mg/l)	C: contact time (min)	D: adsorbent dosage (g/l)	Final MB concentration (mg/l)	Removal (%)
				°		°
1	3	130	10	1.4	130	0
2	11	10	10	1.4	2.309	76.91
3	11	10	50	1.4	1.291	87.09
4	11	130	10	1.4	83.72	35.6
5	7	70	30	1.1	50.02	28.54
6	3	10	50	1.4	1.52	84.78
7	5	70	30	0.8	59.27	15.32
8	7	70	30	0.5	53.55	23.49
9	9	70	30	0.8	57.54	17.8
10	11	130	10	0.2	84.63	34.9
11	11	130	50	0.2	95.11	24.53
12	7	40	30	0.8	35.54	11.15
13	3	130	50	1.4	94.18	27.55
14	11	10	10	0.2	7.66	23.34
15	7	70	40	0.8	55.47	20.75
16	7	70	20	0.8	56.63	19.1
17	3	10	10	0.2	7.88	21.11
18	3	10	50	0.2	3.35	66.41
19	3	10	10	1.4	7.66	23.34
20	11	10	50	0.2	6.96	30.4
21	11	130	50	0.2	130	0
22	3	130	50	0.2	97.95	24.65
23	3	10	10	1.4	7.53	24.7
24	7	100	30	0.8	82.37	17.63
25	7	40	30	0.8	33.02	17.43
26	7	40	30	0.8	31.42	21.65
27	7	40	30	0.8	25.55	36.11
28	7	40	30	0.8	33.01	17.47
29	7	40	30	0.8	32.04	19.89
30	7	40	30	0.8	29.74	25.65

**Table 2:** Data analyzing and modeling.

Source Model	Sum of squares	Df	Mean square	F value	p-Value Prob > F
Model	39413.65	14	2815.26	46.38	<0.0001
A	3051.81	1	3051.81	50.27	<0.0001
B	13336.16	1	13336.16	219.68	<0.0001
C	370.28	1	370.28	6.10	0.0162
D	10729.56	1	10729.56	176.75	<0.0001
AB	969.03	1	969.03	15.96	0.0002
AC	0.44	1	0.44	7.23	0.9325
AD	18.12	1	18.12	0.3	0.5868
BC	44.83	1	44.83	0.74	0.3934
BD	7893.79	1	7893.79	130.03	<0.0001
CD	118.79	1	118.79	1.96	0.1668
A^2^	25.85	1	25.85	0.43	0.5164
B^2^	91.62	1	91.62	1.51	0.2238
C^2^	5.66	1	5.66	0.093	0.7610
D^2^	5.23	1	5.23	0.086	0.7702
Residual	3824.50	63	60.71		
Lack of fit	1384.32	10	138.43	3.01	0.0045
Pure error	2440.18	53	46.04		
Cor total	43238.15	77			
Adeq. Precision	24.59				
R^2^ = 0.91	R^2^(_Adj_) = 0.89	R^2^_(pred)_ = 0.85			

Notes: R^2^: Determination coefficient, R^2^_Adj_: Adjusted R^2^, Adeq. Precision: Adequate precision.

**Table 3:** Optimized values of parameters on the MB removal by AC-CS.

Parameters	Optimized amounts
Adsorbent dosage (g)	1.3
Initial concentration (mg/l)	13.16
pH	8.37
Time (min)	42

## 2 Experimental design, materials, and methods

### 2.1 Materials

Methylene blue (Germany, Merck Company) with high purity (99.9%) has been used for preparing stoke solution (Table 4), 1000 mg/l of methylene blue were dissolved in 1 L of distilled water. In order to homogenize, the solution was placed on a slow-paced stirrer for 1 hour. In order to adjust pH, sulfuric acid (0.1 N) and sodium hydroxide (0.1 N) were used.

**Table 4:** Characteristics and chemical structure of methylene blue.

Parameters	Properties
Molecular formula	C_16_H_18_N_3_SCl
Molecular weight (g/mol)	319.85
λ max (nm)	664
Type of dye	cationic
**Chemical structure**	

### 2.2 Activated carbon preparation

In this study, the adsorbent (corn), as a 1-year-old plant from the suburbs of Kermanshah, was collected and transferred to the laboratory of the School of Public Health. At first, corn stalks were washed and rinsed to remove impurities and dust with distilled water. In the next step, the branches were cut into smaller pieces (5 cm) and were washed by distilled water several times. Then according to the objectives of the study, the required amount of prepared boughs was put in Oven (Memmert 854, Germany) at 150 °C for 3 hours. In order to activate the raw material adjusted method that has been used by Karagöz, S., et al. (2008) as a thermochemical activation was base in this study. At first stage by chemical activation, the stalks were placed in the activating agent (1 normal sulfuric acid) in a weight ratio of 1–10 for 24 hours. Then, the residue of sulfuric acid removed from boughs by washing and exposed to free air for drying. Then carbonization was performed for 1 hour at 500 °C in the electrical furnace (Nabertherm Company, Germany). The final step was to neutralization and drying of activated carbon [5]. The AC after dying has been sieved to obtain mesh of 50 (0.2 mm) and stored in a desiccator.

### 2.3 Modelling and optimization

The effect of process parameters on the adsorbent efficiency has been investigated to remove of dye from the aqueous solution using design of experimental (DOE) software (version 8). This method has the ability to limit systematic errors by estimating the tests so that they can minimize the experiments [6]. At this stage, four independent variables of contact time, absorption dose, pH, and initial concentration of MB are considered as major variables that they are more effective parameters on adsorption process [1]. Optimization of these factors can have a significant effect on the process efficiency and reduce the cost of treatment. Because of this, RSM was used with the use of CCD through the DOE software to create an empirical model and statistical analysis based on the objectives of this study. In addition to the ability of DOE for designing of experiments and perform statistical analysis, this software is also capable of constructing and presenting mathematical models and process optimization [7]. Meanwhile, in addition to the effect of each variable, their interaction effects can also be examined by this method. Based on nature of the absorption process and the necessity of repeating each test, in order to increase the accuracy and validity of the results, three repetitions are considered; therefore, the number of tests was 78. Each of the variables is the response to the concentration of dye (Y) in effluent or the percentage of removal efficiency in the form of a polynomial regression model as an independent function (Equation (1)) [7], [8].

(1) $$\text{Y}={\;\text{β}}_{0}+\sum_{\text{i}=1}^{\text{k}}{\text{β}}_{\text{i}}{\text{x}}_{\text{i}}+\sum_{\text{i}=1}^{\text{k}}{\text{β}}_{\text{ii}}{\text{x}}_{\text{i}}^{2}+\sum_{\text{i}<\text{j}}^{\text{k}}\sum {\text{β}}_{\text{ij}}{\text{x}}_{\text{i}}{\text{x}}_{\text{j}}+\text{e}\;$$

Where y is the predicted response related to each factor level combination; i represents linear coefficient, j stands for the quadratic coefficients, β_0_ is the regression coefficient; and βi, β_ii_, and β_ij_ are linear effect, quadratic effect, and 2-way linear by linear interaction effect, respectively; x_i_ and x_j_ are the coded values of independent variables; k is the number of studied and optimized factors in the experiment, and e is the residual error.

The polynomial regression model was applied between the response variable and the corresponding code values from different process variables (A, B, C and D). Finally, the best equation of the consistent model was obtained based on equation (2).

(2) $$\text{γ}=+\text{21.31}+7.85\text{A}-16.41\text{B}+\text{2.74C}+\text{14.72D}+4.49\text{AB}-0.096\text{AC}+0.61\text{AD}-0.97\text{BC}-12.82\text{BD}+1.57\text{CD}-{7.20\text{A}}^{2}+13.55{\;\text{B}}^{2}{+3.37\text{C}}^{2}{+3.24\text{D}}^{2}$$

In this equation, the values of pH (A), the initial concentration of methylene blue (B), contact time (C) and adsorbent dose (D). Positive coefficients indicate the positive effect of the parameters in the range tested on methylene blue adsorption, which increases the absorption and response rate, and the image of this state can be attributed to values with a negative coefficient [7], [9].

### 2.4 Batch adsorption process

The amount of 100 ml of methylene blue in various concentrations of 10–130 mg/L with the desired dosages of the adsorbent (0.2–1.4 g/L), by adjusting the pH in the appropriate range of 3–11 at a contact time of 10–50 min, have been investigated for the removal of dye. During the process, the temperature was maintained at 25 ± 2 °C. After a specific adsorption period, samples have been centrifuged (Shimifan, Iran) for 5 minutes with 4000 rpm. The supernatant was used directly to determine the absorbance at 675 nm using a spectrophotometer (Jenway 6305, Germany). The measurement and efficiency of the adsorption process by Equation (3) and the adsorption capacity or amount of absorbed dye from the solution per unit of absorbent weight were calculated using Equation (4) [10].

(3) $$\;\text{\%Removal}=\frac{{\text{C}}_{0}-{\text{C}}_{\text{e}}}{{\text{C}}_{0}}\times 100$$

(4) $${\text{q}}_{\text{e}}=\frac{{\text{C}}_{0}-{\text{C}}_{\text{e}}}{\text{w}}\times \text{V}$$
