# Supplementary material for: Dataset on adsorption of methylene blue from aqueous solution onto activated carbon obtained from low cost wastes by chemical-thermal activation – modelling using response surface methodology
Source: Data Brief. 2019 May 23;25:104036. doi: 10.1016/j.dib.2019.104036 (PMC6565604; doi:10.1016/j.dib.2019.104036)
Supplement: Multimedia component 1 [file mmc1.doc]

Conflict of Interest and Authorship Conformation Form

| Author’s name | Affiliation |
| --- | --- |
| Danial Nayeri | Student Research Committee, Kermanshah University of Medical Sciences, Kermanshah, Iran. |
| Seyyed Alireza Mousavi | Department of Environmental Health, faculty of health, and Research Center for Environmental Determinants of Health (RCEDH), Kermanshah University of Medical Sciences, Kermanshah, Iran.  Social Development and Health Promotion Research Center, Kermanshah University of Medical Sciences, Kermanshah, Iran. |
| Mahsa Fatahi | Student Research Committee, Kermanshah University of Medical Sciences, Kermanshah, Iran. |
| Ali Almasi | Department of Environmental Health, faculty of health, and Research Center for Environmental Determinants of Health (RCEDH), Kermanshah University of Medical Sciences, Kermanshah, Iran.  Social Development and Health Promotion Research Center, Kermanshah University of Medical Sciences, Kermanshah, Iran. |
| Faranak Khodadoost | Student Research Committee, Kermanshah University of Medical Sciences, Kermanshah, Iran. |
